# Exhaled Nitric Oxide in Children with Asthma and Short-Term PM_2.5_ Exposure in Seattle

**DOI:** 10.1289/ehp.7883

**Published:** 2005-08-08

**Authors:** Therese F. Mar, Karen Jansen, Kristen Shepherd, Thomas Lumley, Timothy V. Larson, Jane Q. Koenig

**Affiliations:** 1 Department of Environmental Health and Occupational Sciences,; 2 Department of Biostatistics, and; 3 Department of Civil and Environmental Engineering, University of Washington, Seattle, Washington, USA

**Keywords:** airway inflammation, asthma, children, exhaled nitric oxide, particulate matter less than or equal to 2.5 μm, short-term exposure

## Abstract

The objective of this study was to evaluate associations between short-term (hourly) exposures to particulate matter with aerodynamic diameters < 2.5 μm (PM_2.5_) and the fractional concentration of nitric oxide in exhaled breath (Fe_NO_) in children with asthma participating in an intensive panel study in Seattle, Washington. The exposure data were collected with tapered element oscillation microbalance (TEOM) PM_2.5_ monitors operated by the local air agency at three sites in the Seattle area. Fe_NO_ is a marker of airway inflammation and is elevated in individuals with asthma. Previously, we reported that offline measurements of Fe_NO_ are associated with 24-hr average PM_2.5_ in a panel of 19 children with asthma in Seattle. In the present study using the same children, we used a polynomial distributed lag model to assess the association between hourly lags in PM_2.5_ exposure and Fe_NO_ levels. Our model controlled for age, ambient NO levels, temperature, relative humidity, and modification by use of inhaled corticosteroids. We found that Fe_NO_ was associated with hourly averages of PM_2.5_ up to 10–12 hr after exposure. The sum of the coefficients for the lag times associated with PM_2.5_ in the distributed lag model was 7.0 ppm Fe_NO_. The single-lag-model Fe_NO_ effect was 6.9 [95% confidence interval (CI), 3.4 to 10.6 ppb] for a 1-hr lag, 6.3 (95% CI, 2.6 to 9.9 ppb ) for a 4-hr lag, and 0.5 (95% CI, −1.1 to 2.1 ppb) for an 8-hr lag. These data provide new information concerning the lag structure between PM_2.5_ exposure and a respiratory health outcome in children with asthma.

Most studies of relationships between particulate matter (PM) air pollution and health are based on 24-hr PM measurements. This approach has been driven mainly by the availability of 24-hr gravimetric monitors operated by the U.S. Environmental Protection Agency. However, there currently are several continuous PM monitors in use for documenting community exposure, and these data allow investigators to ask questions about very short-term (hourly) lags between health outcomes and PM exposure. It is important to understand the interval between exposure and health event (lag) as fully as possible because this may help our understanding of both the mechanisms underlying the event and the source of the PM.

Nitric oxide levels in airways are suggestive of the degree of airway inflammation and injury (Yates 2001; Bates and Silkoff 2003). The fractional concentration of NO in exhaled breath (Fe_NO_) is easy to measure in exhaled breath and is a noninvasive lung measurement used to diagnose asthma (Jones et al. 2001; Kharitonov and Barnes 2000; Zeidler et al. 2004). Fe_NO_ is elevated in subjects with asthma, is elevated during an asthmatic attack (Jones et al. 2001; Silvestri et al. 2001; Yates 2001), and is reduced when subjects with asthma are treated with anti-inflammatory medications such as inhaled corticosteroids (ICS) (Beck-Ripp et al. 2002). Recently, we reported an association between 24-hr average PM with aerodynamic diameters < 2.5 μm (PM_2.5_) and Fe_NO_ in children with asthma participating in a panel study in Seattle, Washington (Koenig et al. 2003). We observed an approximately 4-ppb average increase in Fe_NO_ for a 10-μg/m^3^ increase in PM_2.5_. Earlier studies also found that community outdoor air was associated with changes in Fe_NO_ (Van Amsterdam et al. 1999, 2000). More recently, Fe_NO_ has been associated with PM exposure in adults with cardiovascular and respiratory disease in Steubenville, Ohio (Adamkiewicz et al. 2004) and in adults with respiratory disease in Seattle (Jansen et al. 2004). The Steubenville study evaluated short-term exposures using moving-average data to reflect cumulative exposures. They reported associations between cumulative average PM_2.5_ up to 12 hr before the Fe_NO_ measurement (Adamkiewicz et al. 2004).

The objective of this study was to compare short-term (hourly) exposures to PM with Fe_NO_ concentrations in children with asthma and to compare these short-term results with the earlier results. Our hypothesis was that short-term lags would show stronger associations with Fe_NO_ than would 24-hr average lags. Defining the most likely interval between exposure and Fe_NO_ response would be useful for designing future studies.

## Materials and Methods

This research was part of an intensive exposure assessment and health effects panel study of susceptible subpopulations in Seattle from 1999 through 2002 (Koenig et al. 2003; Liu et al. 2003). Nineteen children, 6–13 years of age, were recruited from a local asthma and allergy clinic. All had physician-diagnosed asthma and were prescribed asthma medications daily or regularly. Each subject in the panel was asked to participate for a 10-day monitoring session in the winter of 2000–2001 and the spring of 2001. Fourteen children participated in the Fe_NO_ study during the winter heating season, and 15 children participated during spring. Ten participated in both seasons. Approximately half of the children were prescribed ICS therapy. The remainder was prescribed only inhaled albuterol as needed.

### Exposure data.

Hourly PM_2.5_ data were collected at three fixed sites within the Seattle area by the local air agency with tapered element oscillating microbalances (TEOMs; Rupprecht and Patashnick Co./Thermo Electron, East Greenbush, NY). Descriptive statistics on covariate measurements are given in Table 1.

**Figure 1:**
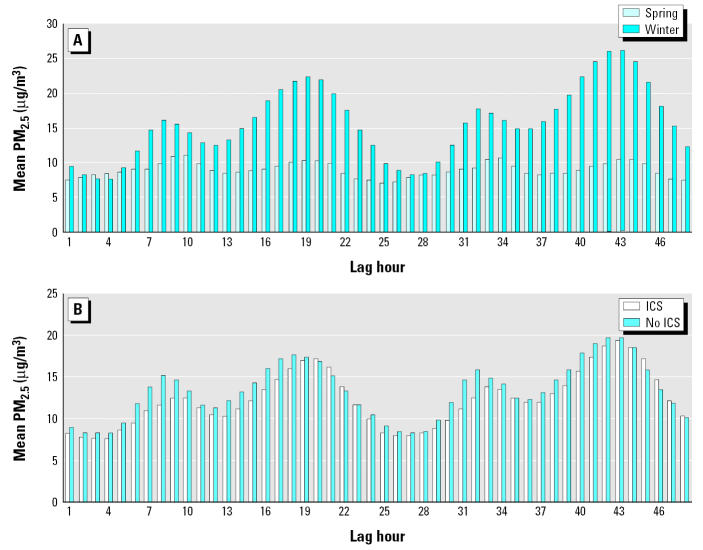
Comparison of mean PM_2.5_ for all subjects stratified by season (*A*) or ICS medication use (*B*).

**Figure 2:**
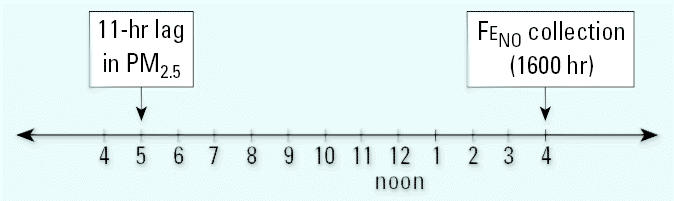
Schematic of real-time and hourly lags (0400 hr to 1600 hr) in PM_2.5_ relative to Fe_NO_ collection.

The average concentration of PM_2.5_ from the TEOM monitors for all subjects stratified by season and ICS use are shown in Figure 1. Average PM_2.5_ concentrations vary with exposure lag. PM_2.5_ concentrations are higher in the winter sessions compared with spring sessions, with winter peaks occurring in the late evening/early morning hours (Fe_NO_ measurements were taken at or about 1600 hr Pacific standard time; see Figure 2). There is little difference in PM_2.5_ exposure between ICS users and nonusers.

### Exhaled NO.

Fe_NO_ was collected as described in a previous report (Koenig et al. 2003). Briefly, all children participated for 10 continuous days of air pollution monitoring and health measurements. Exhaled breath was collected in a Mylar balloon at approximately 1600 hr each day using an offline Fe_NO_ protocol. Exhaled breath was measured with a chemiluminescent nitrogen oxide analyzer (model 200A; API, San Diego, CA). Children were asked to refrain from eating for 1 hr before the exhaled breath collection. Pulmonary function testing was conducted after the exhaled breath because a deep inspiration may affect Fe_NO_ values (Deykin et al. 1998). Subject characteristics and Fe_NO_ measurements are presented in Table 2.

### Statistical analysis.

We assessed the association between short-term effects of particulate air pollution and Fe_NO_ using a polynomial distributed lag (pdl) model for PM_2.5_ up to 48 hr after exposure. The pdl model allows air pollution effects at many different lags to be estimated in the same model. The model assumes that the air pollution effect varies smoothly with lag, and approximates this smooth variation by a polynomial curve. The pdl model with 3 degrees of freedom is estimated by Poisson regression using a transformed set of three exposure variables that are not highly collinear. The three estimated coefficients specify the polynomial curve, which in turn gives associations at all lags. In addition to estimating the air pollution effect over many lags, the model can be used to estimate the total air pollution effect by summing the estimates at each lag (Schwartz 2000).

Pdl models are used with time-series data where the effects of a regressor are distributed over time. This type of model constrains the coefficients to follow a polynomial that reduces the number of parameters and therefore reduces the effects of collinearity in the lag variables. Similar models have been used to look at the effect of daily lags in air pollution exposure and mortality (Goodman et al. 2004; Schwartz 2000).

Equation 1 describes the model that was used for the analysis. Each pollution variable was modeled as a difference between the daily PM_2.5_ level and the average exposure of the subject during his or her session because we are primarily interested in a within-subject, within-session effect. This model also included a term to account for the ambient concentrations of NO that could potentially contaminate our Fe_NO_ measurements. Koenig et al. (2003) used a similar model to look at the within-subject effects of daily increases in PM_2.5_ and Fe_NO_. Model estimates were obtained using the linear mixed-effect equations and the generalized least squares (GLS) estimator in Stata (version 6.0; StataCorp, College Station, TX). As a sensitivity analysis, model estimates were also obtained using a generalized estimating equations (GEE) with an exchangeable working correlation matrix and robust standard errors.


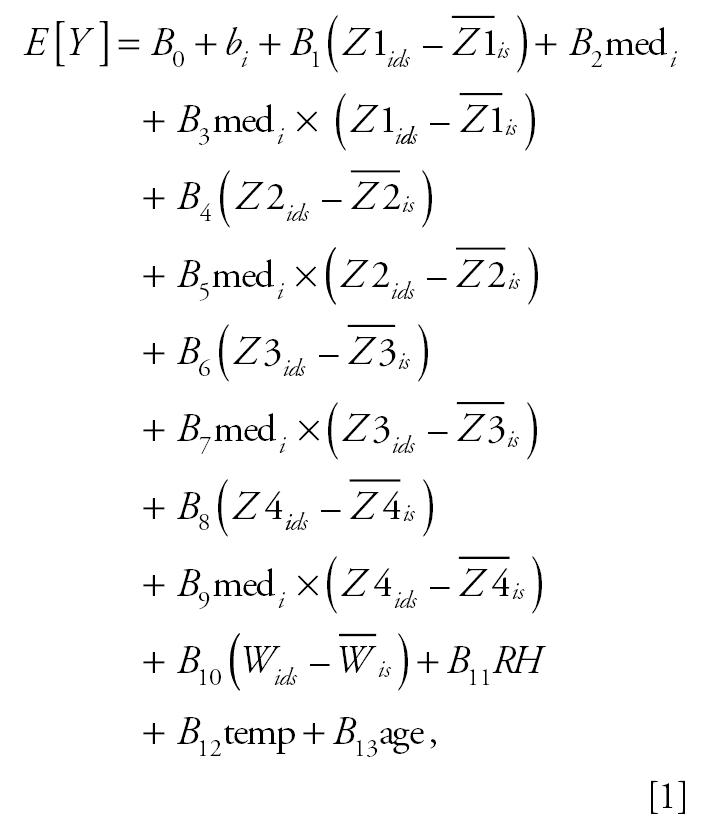


where


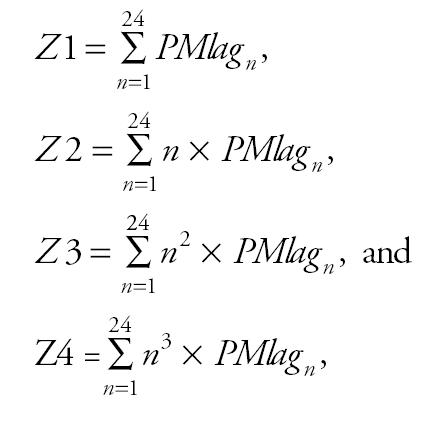


*W* is the ambient NO concentration, *ids* is the PM reading for individual *i* on day *d* during session *s*, *is* is the mean PM reading for a subject during a session, *i* is the mean PM reading for a subject during all of their sessions, med*_i_* is an indicator variable for medication use (constant for each subject), and *RH* is relative humidity.

The coefficients for each lag term were obtained using


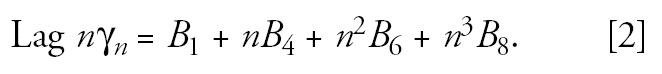


## Results

**Figure 3:**
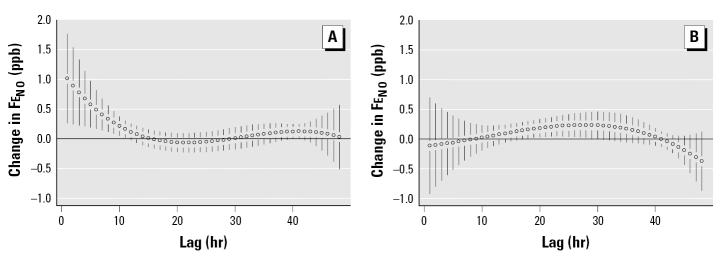
Change in Fe_NO_ per 10-μg/m^3^ increase in PM_2.5_ (*A*) in subjects not prescribed ICS and (*B*) in subjects prescribed ICS therapy. TEOM readings were averaged from three central sites (Lynnwood, Lake Forest Park, and Kent) for hourly lags from 1 to 48. Model adjusted for temperature, relative humidity, and age. One-hour averaged PM_2.5_ concentrations ranged from 8.3 μg/m^3^ at 3-hr lag to 15.2 at 8-hr lag, suggesting that short time-lag periods rather than peak values may determine this health outcome. Error bars indicate 95% confidence intervals.

The results of the polynomial distributed model for the short-term effect of PM_2.5_ on Fe_NO_ in subjects not taking ICS are shown in Figure 3A. Significant increases in Fe_NO_ associated with PM_2.5_ can be observed in the first 11 hr after exposure. There is also some suggestion of an increase in Fe_NO_ between 38 and 41 hr after exposure. The overall effect of a prolonged exposure to PM_2.5_ is obtained by summing up the estimated effects at each time lag. The sum of all the lag coefficients (β) over 48 hr was 7.0 ppb Fe_NO_ per 10-μg/m^3^ increase in PM_2.5_.

The short-term effects of PM_2.5_ on Fe_NO_ for subjects who were prescribed ICS medications are shown in Figure 3B. In general, we found no association between Fe_NO_ and PM_2.5_ in subjects prescribed ICS. However, a very small association was observed from the 18-hr lag to the 30-hr lag. This small increase in Fe_NO_ (ranging from 0.16 to 0.23 ppb per 10-μg/m^3^ increase in PM_2.5_) would not be of clinical significance. For ICS users, the overall effect of PM_2.5_ over 48 hr is a 0.3-ppb increase in Fe_NO_ per 10-μg/m^3^ increase in PM_2.5_.

The association between Fe_NO_ and PM_2.5_ averaged over 1 hr at various lags was also analyzed in a single-lag, linear mixed-effects regression model. These results are shown in Table 3. With the single-lag model where PM_2.5_ was averaged over 1 hr, we found that 7.0-ppb and 6.3-ppb increases in Fe_NO_ were associated with PM_2.5_ lagged 1 and 4 hr, respectively, in subjects not taking ICS. No association was found in subjects taking ICS. No associations were found with a PM_2.5_ exposure 8 hr previous in either group of children (Table 3).

**Figure 4:**
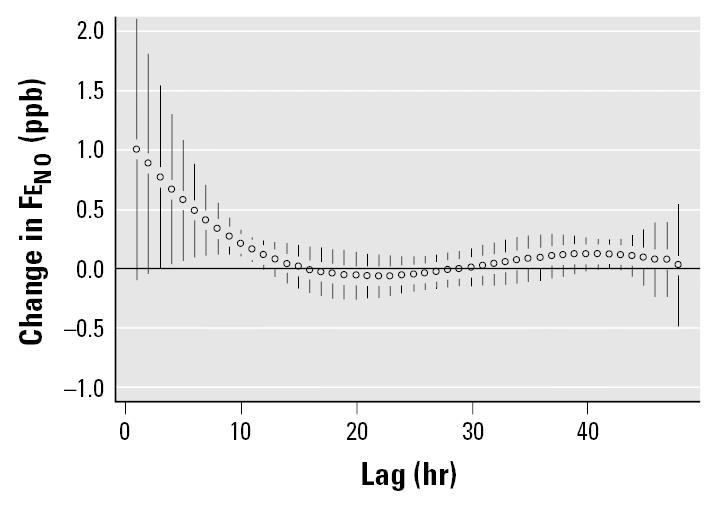
Change in Fe_NO_ per 10-μg/m^3^ increase in PM_2.5_ in subjects not prescribed ICS therapy. TEOM readings averaged from three sites using GEE model. Error bars indicate 95% confidence intervals.

We also tested for the lag structure in these data using a GEE model that controls for autocorrelations in the data (Figure 4). The distributed lag pattern was similar to that with the linear-effects model; however, associations between Fe_NO_ and PM_2.5_ dropped out for the earliest hourly lags (exposures at 1 and 2 hr before breath collection).

## Discussion

The objective of this study was to evaluate the temporal relationship between prior exposure to PM_2.5_ and increases in Fe_NO_ in the airways of children with asthma. Using a pdl model, we found that Fe_NO_ was associated with hourly averaged PM_2.5_ exposure up to 10–12 hr before the health measurement in subjects not prescribed ICS. The overall effect was a 7-ppb increase in Fe_NO_ associated with a 10-μg/m^3^ increase in PM_2.5_ relative to each subject’s mean PM_2.5_ exposure.

The advantage of using the pdl model is the ability to reduce the collinearity in the individual lags, allowing a better understanding of the relative contribution of individual lags and, in this case, the short-term effect of PM_2.5_ exposure on Fe_NO_. The similarity in results from the analyses using the linear-effects model with the GLS estimator and those using the GEE model strengthens our confidence in these results (Table 3). It is apparent from Figure 1 that associations between PM_2.5_ and Fe_NO_ during the 48-hr period of analysis were not predicted by the average PM_2.5_ concentration during that period, but rather by exposures up to 11 hr before Fe_NO_ collection. These results are dependent on the pdl model used; different models (e.g., first- and second-degree pdl) may show associations with slightly different time patterns.

Additionally, using a single lag at specific time periods (1, 4, and 8 hr before Fe_NO_ collection) for the children not prescribed ICS, we found a 7-ppb increase in Fe_NO_ for a 10-μg/m^3^ increase in PM_2.5_ exposure 1 hr earlier and a 6.3-ppb increase associated with an 10-μg/m^3^ increase in PM_2.5_ 4 hr earlier. The estimate of Fe_NO_ increase is similar to that seen in the pdl model; however, the multiple-hour curve gives more complete information. The limitation of using a single-lag model is that the estimated PM_2.5_ effect at each of the lag hours could be confounded by the effect of other lag hours. Our single-lag model was based on 1 hr averaged PM_2.5_ rather than a running average of PM_2.5_ for a cumulative exposure effect. Although the pdl model is the preferred model, both the single-lag and the pdl models resulted in similar effect estimates. The results from our study are consistent with those reported by Adamkiewicz et al. (2004), who found increases in Fe_NO_ significantly associated with PM_2.5_ exposures up to 12 hr previously. That study, however, used individual hourly lag models.

The results from our analysis using the third-degree pdl model indicate that the effect of PM_2.5_ on Fe_NO_ is not just immediate but may have an effect up to 11 hr after exposure. Because in our study Fe_NO_ was measured at approximately 1600 hr each day, this would indicate that PM_2.5_ exposure from 0500 hr to 1600 hr (the time of Fe_NO_ measurement) is the relevant period of exposure. Using our time line, this would suggest that sources that predominate during daytime hours are most important.

This is one of the first studies to report short-term temporal relationships between PM_2.5_ and health outcomes in children with asthma. In another short-term study, hourly averages of PM were associated with respiratory symptoms in children with asthma (Delfino et al. 1998). More recently, that group, using personal monitors, reported that associations between PM and lung function derived from 1- or 8-hr PM_2.5_ averages did not differ from associations based on 24-hr averages (Delfino et al. 2004). These findings add more information about the relationship between PM exposure and respiratory effects and may be useful for clinicians and patients. This information also may be informative for researchers in their experiment design efforts.

The relatively wide range of exposure lags associated with increased Fe_NO_ in children with asthma that we observed suggests that more than one mechanism may be underlying changes in respiratory NO induced by air pollution. Rapid responses are associated with nervous system changes through nerve receptors or synaptic mediators, whereas delayed responses are sometimes attributed to up-regulation of gene expression and enzyme synthesis. These actions are compatible with up-regulation of NO, which has several roles in the lung (Deykin and Kharitonov 2003). Coincidentally, a recent study of allergen challenges in subjects with asthma found that Fe_NO_ was initially decreased after exposure but increased 48 hr after exposure (Ricciardolo et al. 2003). Perhaps air pollution interactions in the airways differ from those of proteins such as allergens.

In conclusion, in this study we present additional data for the use of lag structure selection in epidemiologic studies of air pollution, an area that has received considerable attention. Future studies using sequential measurements of Fe_NO_ will allow us to better identify the sources of and mechanisms underlying this health outcome.

## Figures and Tables

**Table 1:** Summary statistics for daily averages of temperature, relative humidity, and ambient NO.

	Minimum	Maximum	Mean ± SD
Temperature (°F)	33	68.7	44.5 ± 6.5
Relative humidity (%)	55.3	96.5	78.6 ± 10.1
Ambient NO (ppb)	0.003	0.099	0.018 ± 0.023

**Table 2:** Age and Fe_NO_ values stratified by age, sex, and medication use.

			Fe_NO_		
	No.	Age (mean ± SD)	Minimum	Maximum	Mean ± SD
Sex					
Female	5	11.2 ± 1.3	5	48.1	13.3 ± 6.3
Male	14	8.2 ± 1.7	5.3	79.8	16.2 ± 10.7
Medication use					
ICS	9	9.7 ± 1.4	5.3	79.8	12.7 ± 7.7
No ICS	10	8.3 ± 2.4	5	72.1	18.4 ± 11.0

**Table 3:** Short-term effects of air pollution on Fe_NO_ from the linear-effects model.

Metric	Medication use	Change in Fe_NO_	95% Confidence interval	*p*-Value
1-hr lag	No meds	6.99	3.43 to 10.55	0
	Meds	−0.18	−3.33 to 2.97	0.911
4-hr lag	No meds	6.30	2.64 to 9.97	0.001
	Meds	−0.77	−4.58 to 3.04	0.691
8-hr lag	No meds	0.46	−1.18 to 2.11	0.58
	Meds	0.40	−1.94 to 2.74	0.736
